# Immunocontraception for Managing Feral Cattle in Hong Kong

**DOI:** 10.1371/journal.pone.0121598

**Published:** 2015-04-09

**Authors:** Giovanna Massei, Ka-Kei Koon, Steven Benton, Richard Brown, Matt Gomm, Darcy S. Orahood, Stéphane Pietravalle, Douglas C. Eckery

**Affiliations:** 1 National Wildlife Management Centre, Animal and Plant Health Agency, York, United Kingdom; 2 Agriculture, Fisheries and Conservation Department, Animal Management Division, Hong Kong SAR, China; 3 The School of Veterinary Medicine, City University of Hong Kong, Hong Kong SAR, China; 4 USDA APHIS National Wildlife Research Center, Fort Collins, Colorado, United States of America; 5 Food and Environment Research Agency, York, United Kingdom; Thomas Jefferson University, UNITED STATES

## Abstract

Conflicts between human interests and feral cattle in Hong Kong derive from growing numbers of free-roaming cattle. Public antipathy towards lethal population control led the local authorities to consider fertility control to reduce cattle numbers. This study assessed the potential side effects of the immunocontraceptive GonaCon on individual female cattle and established the effectiveness of GonaCon to induce infertility. We evaluated GonaCon in 34 captive cattle assigned to four groups: Control administered a sham solution; Webbed (surgically sterilized through removal of the oviducts), administered one dose of GonaCon; Webbed, administered one dose of GonaCon and a booster dose three months later, and Treated, administered one dose of GonaCon. The side effects of GonaCon were assessed by monitoring injection site, body weight, body condition, size of lymph nodes, body temperature, and feeding behaviour 1 week and 1, 3, 6, 9 and 12 months after vaccination and by haematological and biochemical variables at vaccination and three months post-vaccination. The effectiveness of GonaCon to cause infertility was monitored by quantifying anti-GnRH antibody titres and by using kits to detect cycling and pregnancy. GonaCon-treated cattle showed no injection site reaction, limping, or abnormal behaviour. No differences were observed in all physiological and welfare indicators between control and vaccinated cattle. All control cattle and 4 of the 12 cattle in the Treated group became pregnant. Cattle administered a booster dose had higher anti-GnRH antibody titres than cattle that received one dose. We concluded that GonaCon does not compromise the animals’ welfare and is effective in reducing fertility in cattle. A booster dose is likely to increase the duration of infertility. Further studies are required to assess the feasibility and costs of immunocontraception for controlling free-roaming cattle populations.

## Introduction

Conflicts between human activities, wildlife and feral animals are often associated with overabundant or expanding animal populations and are likely to increase in the near future [1–3]. Culling has been used traditionally to mitigate these conflicts. However, public antipathy to culling has grown because of concerns about animal welfare, human safety in urban settings, and environmental impact of some control method [4–7]. Opposition to lethal methods, particularly strong for high profile, iconic species, has promoted interest in alternative options, such as translocation and fertility control, to manage overabundant animal populations [8–11]. Translocations of problem animals, often perceived as the best management option, is expensive and may have a number of other drawbacks that make this method often unsuitable to resolve human-wildlife conflicts [12–16].

Fertility control through contraception may offer a humane and effective means of managing overabundant animal populations [9, 10, 17]. Single-shot, injectable immunocontraceptive vaccines are increasingly considered as potential options for controlling size and growth of wildlife populations [18–20]. These vaccines act by inducing antibodies to proteins or hormones essential for reproduction. For instance, gonadotropin-releasing hormone (GnRH)-based vaccines cause the production of antibodies to GnRH, thus preventing the production of sex hormones and ultimately inhibiting ovulation. One of these vaccines is the single-dose injectable GnRH vaccine GonaCon, shown to decrease significantly fertility for at least 1–6 years in a wide spectrum of species including deer, wild boar and feral pigs (*Sus scrofa*), cats (*Felis catus*), horses (*Equus caballus*), and bison (*Bison bison*) [17, 21–24]. As GonaCon prevents ovulation, treated females do not exhibit oestrous for several years following vaccination [25]. In some species, vaccination with GonaCon was observed to cause an apparently non-painful granuloma or a sterile abscess at the injection site. For instance cats treated with GonaCon had a palpable non-painful injection-site granuloma [26] and both white-tailed deer (*Odocoileus virginianus*) and elk (*Cervus elaphus*) had granulomatous nodules and sterile abscesses at injection sites and in lymph nodes, although no evidence of limping or impaired mobility was observed in these animals [27–28]. Conversely, no adverse effects were observed in GonaCon-treated wild boar, prairie dogs (*Cynomys ludovicianus)*, and wild horses [17, 23, 24, 29]. As the potential side effects as well as the effectiveness of GonaCon, in terms of both proportion of animals rendered infertile and duration of infertility, vary among species, trials under controlled conditions are advisable when this vaccine is tested on a new species.

**Fig 1:**
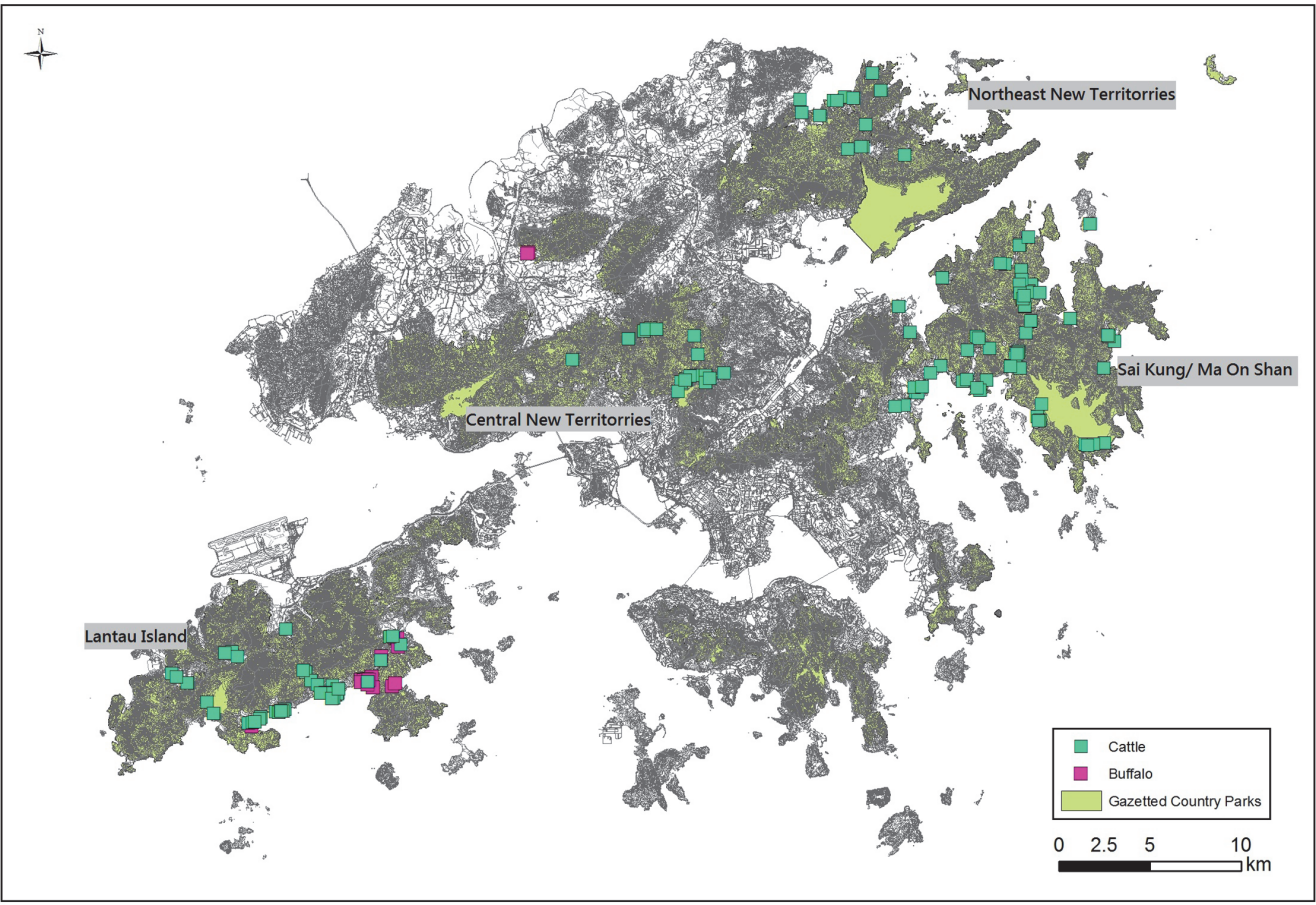
Location of the main populations of feral cattle (green square symbols) in Hong Kong.

In the unique setting of Hong Kong, an estimated 1250 South China feral cattle (*Bos taurus/ Bos indicus*) exist as free-living animals [30, 31]. These cattle have not been actively managed since they were released by local farmers over four decades ago, when the decline of agricultural activities meant they were no longer needed as draught animals. For a variety of reasons, including religious beliefs, many farmers did not take the cattle to slaughter but simply let them wander away freely. At present, most of the cattle are not found in the highly urbanized areas of Hong Kong but they occur in the following four main areas, isolated from each other: Lantau Island, Sai Kung/ Ma On Shan, Central New Territories, and Northeast New Territories (Fig 1). The impacts of feral cattle include traffic disturbance and accidents, environmental nuisance, and crop damage. On the other hand, cattle are regarded as part of the local heritage and some of the Hong Kong residents support non-lethal control to manage this species [31].

In 2011, in response to concerns expressed by stakeholders on the impact of feral cattle on human activities and *vice versa*, the Agriculture Fisheries and Conservation Department (AFCD) set up a designated Cattle Management Team for the long-term management of feral cattle populations. One of the main objectives of AFCD is to achieve a stable cattle population so that humans and feral cattle can co-exist in harmony. Feral cattle are captured and transported to the Ta Kwu Ling Operation Center in Fanling, Hong Kong, where animals are surgically sterilized. Bulls are castrated while females are webbed, i.e. the oviducts are removed. After a short holding period at the Operation Center to ensure animals have recovered from surgery, all sterilized cattle are returned to their natal area or relocated to country parks. As capture and transport of animals is impossible in remote areas, the use of immunocontraception was evaluated as an alternative to surgical sterilization.

The overall aim of the proposed project was to provide initial evidence that immunocontraception could be a safe and effective method to decrease fertility in feral cattle, thus assisting the AFCD in its proactive role to provide tools that could be used for reducing population growth of feral cattle in Hong Kong. The specific aims of this study were: 1) to assess the potential side effects of GonaCon on individual animals, and 2) to establish the effectiveness of GonaCon to induce infertility in feral cattle.

## Methods

The study was carried out at the Ta Kwu Ling Operation Center in Fanling, Hong Kong. The center is owned by the Agriculture, Fisheries and Conservation Department (AFCD) that authorized the study. At the beginning of the study, 12 cows that had been surgically sterilized in March 2012 through removal of the oviducts (referred to as “webbing”) were available at the farm. In addition, 22 cows captured between April 2012 and February 2013 were kept for the trial. Animals were kept in a 2025 m^2^ paddock and provided with one bale of hay per 3 cows per day and water *ad libitum*.

All animals were de-wormed with ivermectin 0.5% pour-on (Noromectin Pour-on; Norbrook; Station Works, Newry, Co. Down, N. Ireland, BT35 6JP) and vaccinated with two doses of clostridial vaccine (Ultravac7 in 1; Pfizer; 38–42 Wharf Rd, West Ryde NSW, Australia) 4–6 weeks apart. All cattle resulted negative for brucellosis when tested with the Rose Bengal Test by the AFCD Veterinary Laboratory (in Lin Tong Mei, Sheung Shui, New Territories, HK). All cattle were subjected to a standard screening test for bovine tuberculosis (the Single Intradermal Comparative Cervical Tuberculin test) and all were non-reactors.

On arrival at the farm, each cow was equipped with a plastic, numbered ear-tag for individual identification. The age of the animals, assessed by tooth eruption and replacement, ranged from 18 months to over 4 years old [32]. Cattle fertility was assessed through behavioural observation to detect oestrous cycling or through rectal palpation to determine whether animals were pregnant. For cows that were not observed cycling, Kamar Heatmount Detectors (Kamar Products Inc., 1821 Kamar Plaza 3, Streamboat Springs, CO 80487, USA; hereafter referred as “Kamars”), were glued onto the cow’s tailhead and used to assist in the detection of oestrus. Kamars, commonly employed in farming practice, change colour when a cow is mounted by a conspecific: this generally indicates that the cow that has been mounted is cycling. Out of the 34 cows used for the study, 29 were confirmed to be cycling, 1 was in the last month of pregnancy and the remaining 4 were confirmed to be non-pregnant by rectal palpation.

At the beginning of the study, animals were randomly assigned to treatment groups; the pregnant cow was assigned to treatment with the vaccine. The 4 cows not observed cycling were equally allocated to treatment with vaccine (n = 2) and to control (n = 2) groups.

Cows were assigned to the following groups:

1. Control: treated with saline (C, n = 10)
2. Webbed: treated with a single dose of GonaCon (W, n = 6)
3. Webbed and boosted: treated with a primary dose of GonaCon followed by a booster dose administered three months after the primary dose (WB, n = 6)
4. Treated with a single dose of GonaCon (T, n = 12).

All cattle injected with GonaCon were used to assess potential injection site reaction to the vaccine and immune response to the vaccine. Webbed cattle were used to assess the anti-GnRH antibody response to a single dose or primary and booster doses of the vaccine. Cattle in the Treated group were used to assess the anti-GnRH antibody response to the vaccine and test the ability of GonaCon to induce infertility.

GonaCon, containing 1000 ug GnRH-mollusk-hemocyanin conjugate per ml, was produced at the USDA, National Wildlife Research Center (Fort Collins, CO). On 16 and 17 April 2013 (hereafter referred as vaccination day), each animal was immobilized in a cattle crush and administered 3 ml (i.e. 3000 μg) of GonaCon (Groups W, WB and T) or 3 ml of 0.9% saline solution (Group C) by intra-muscular injection in the left side of the neck using an 18G 40mm needle. Three months after vaccination, cattle of Group WB were administered a 1 ml (i.e. 1000 μg) booster dose of GonaCon. An area of 2–3 cm in diameter was shaved at the injection site to facilitate monitoring of possible reactions to vaccination. For each animal, the following data were collected: 1) Body weight, 2) Age, 3) Body condition score, 4) Size of pre-scapular (PS) and pre-crural (PC) lymph nodes (only on right side due to operator’s safety and handling constraints), and 5) Body temperature. Pictures and the size of the right and left horn of each animal were also collected for identification purposes. Body condition scores were assigned on a scale from 1 to 5, with 1 being the poorest condition, 3 the ideal score, and 5 indicating overweight [33]. The diameter of the PS and PC lymph nodes was estimated by palpation, with two veterinarians taking independent measurements that were then averaged for each animal.

Blood samples were collected on vaccination day and 3, 6, 9 and 12 months post-vaccination. For each cow, blood was collected in two untreated Vacutainers and one EDTA-treated Vacutainer (BD Vacutainers, Belliver Ind. Estate, Plymout, PL6 7BP, UK) for health profile analysis and for anti-GnRH antibody assays. The sera used for anti-GnRH antibody assays were stored at -20° C until assayed.

Six months after vaccination, two bulls of proven fertility were housed with the cows and all females were fitted with Kamars. Observations of Kamars were carried out 5 days per week for 8 weeks to determine the number of cattle that were cycling. Two months later, the bulls were removed from the paddock. A Pregnancy-Associated-Glycoprotein assay (PAG), routinely used as an indicator of cattle pregnancy from day 28 [34], was employed to determine the number of pregnant cattle in Groups T and C one month after the bulls had been removed. Twelve weeks after the bulls had been removed, the reproductive status of all cows was confirmed through internal palpation to detect pregnancy. Animals that were found to be pregnant were administered prostaglandins to induce early-pregnancy abortion.

The study was approved in the UK by the Food and Environment Research Agency Ethical Review Process (ERP, 15/11/2012). As the AFCD in Hong Kong is a government department and not primarily a research institution, it does not have a permanent named IACUC: for this reason, an *ad hoc* Ethic Review Panel was set up specifically for this trial.

### Effects of the GnRH vaccine on behaviour and physiology

The potential side effects of GonaCon on animal health were assessed by post-vaccination monitoring of body weight, haematology and serum biochemistry, lymph node size, body temperature, feeding behavior, and injection site reaction. Data on haematology and serum biochemistry were collected on vaccination day and three months after vaccination. Data on all the other variables were collected on vaccination day and 1 week, and 1, 3, 6, 9 and 12 months post- vaccination. Feeding behavior was monitored by direct observation of cattle, by recording whether animals fed on the hay provided in the morning. Concurrently, the injection site was observed for signs of reaction to vaccination. Injection site reaction was also monitored 1 week, and 1, 3, 6, 9 and 12 months post-vaccination. This was done by applying pressure with one hand on the injection site and recording the animal’s reaction (recorded as “no reaction” or “recoiling from pressure”).

The following biochemical and haematological parameters were provided by Path Lab, Medical Laboratories Ltd (Wan Chai, HK), based on blood collected at the time of vaccination and three months later: total protein, albumin, globulin, aspartame aminotransferase (AST), gamma globulins, phosphokinase (CPK), urea, sodium, calcium, neutrophils, lymphocytes, monocytes, eosinophils, red blood cell count (RBC), haemoglobin, haematocrit, mean corpuscular volume (MCV), mean corpuscular haemoglobin (MCH), mean corpuscular haemoglobin concentration (MCHC), red cell distribution width (RDW), mean platelet volume, and platelet count.

### Effectiveness of the GnRH vaccine to induce infertility

The effectiveness of the vaccine to induce infertility was determined by: 1) Immune response to the vaccine, assessed by measuring serum anti-GnRH antibodies, 2) detection of cycling through the use of Kamars, and 3) confirmation of pregnancy through the PAG assay and internal palpation carried out on all T and C cattle.

An enzyme-linked immunosorbent assay (ELISA) was used to measure anti-GnRH antibody titres. Wells of 96-well plates were coated by adding 200 ng of GnRH-ovalbumin conjugate in 50 μL of carbonate-bicarbonate buffer to each well and incubating overnight. Plates were blocked with a solution of 20% SeaBlock (Thermo Fisher Scientific; Waltham, Massachussets, USA) and 5% Tween 20 in 0.01 M phosphate buffered saline (PBS). Fifty μL of cattle sera, pre-diluted 1:1000 in BUF039 sample diluent (AbD Serotec; Oxford, UK) were serially diluted to 1:256,000 in PBS. A rabbit anti-bovine IgG antibody conjugated to horseradish peroxidase (HRP) was used as the secondary antibody. Tetramethylbenzidine was used as a chromogenic substrate and 2 M sulfuric acid was used to stop the reaction. The optical density (OD) of each sample was measured at 450 nm with a Dynatech MR 5000 microplate reader. The limit of detection (LOD) was estimated as 3 Standard Deviations (SD) of the mean OD for 8 wells included on every plate that were incubated with buffer instead of cow serum; OD values below the LOD were not considered for titre assignment. Titre positive/negative thresholds were established for each dilution factor as the mean of all pre-vaccination ODs plus 2 SD of that mean. Anti-GnRH antibody titres were determined by comparing post-vaccination sample ODs for each animal to the threshold values, and expressed as the highest dilution factor at which the post-vaccination sample OD was greater than the corresponding threshold value.

### Statistical analyses

Differences in body weight between groups were analyzed by a repeated measures analysis of covariance. The initial weight, recorded at vaccination, was used as a covariate. As pregnancy would have affected body weight, from nine months post-vaccination onwards, this variable was compared between groups 1 week and 1, 3 and 6 months post-vaccination.

Repeated measures analysis of covariance was also used to test between group differences in body temperature, body condition score, and size of PS and PC lymph nodes 1 week, and 1, 3 and 6 months post-vaccination.

Differences in biochemistry and haematology among groups were tested through a multivariate analysis of variance (MANOVA) carried out on vaccination day and three months post-vaccination.

A repeated measure analysis of variance was carried out on log-transformed anti-GnRH antibody titres to determine whether the booster dose had an effect on the titres. Anti-GnRH antibody titres were compared for the T, W, and WB treatment groups 3, 6, 9 and 12 months post-vaccination.

A Mann-Whitney test was used to determine whether anti-GnRH antibody titres in Group T could be used to predict infertility. Anti-GnRH antibody titres at 6 months post-vaccination were compared between cattle that were confirmed pregnant nine months post-vaccination and animals that remained infertile nine months post-vaccination.

A McNemar test was used to test the agreement between the detection of cycling assessed by using Kamars, which indicated the likelihood of an animal to become pregnant, and the actual pregnancy status derived from the PAG test and from internal palpation.

A two sample binomial test was used to assess the effectiveness of the immunocontraceptive to induce infertility by comparing the proportion of pregnant versus non-pregnant cattle in Groups T and C. All data analyses were carried out in GenStat 16.1 [35].

## Results

The female in Group T that was already pregnant at the time the study was initiated gave birth to a calf two days after vaccination and was thereafter monitored with the rest of the group. One animal in Group C showed a sudden onset of ataxia about a month after vaccination and was euthanized on humane ground. The carcass was sent to lab for post mortem examination and none of the histopathological changes explained the condition. Thus nine animals remained in Group C.

All cattle were observed eating normally following primary vaccination and, when applicable, following administration of the booster dose. Of the 24 cows treated with GonaCon, one animal in Group W showed a mild injection site reaction as diffuse swelling at one week post-vaccination. This reaction was observed again in the same animal one month later but disappeared thereafter. Another animal in Group W had a lump, 1 cm in diameter, at the injection site 12 months after vaccination. In both instances, the animals did not react when a researcher put pressure on the injection site, suggesting the swelling was not painful. None of the other cattle showed any injection site reaction, limping, or abnormal behaviour that might have indicated suffering or discomfort. It was concluded that GonaCon did not cause any obvious, persistent injection site reaction.

**Fig 2:**
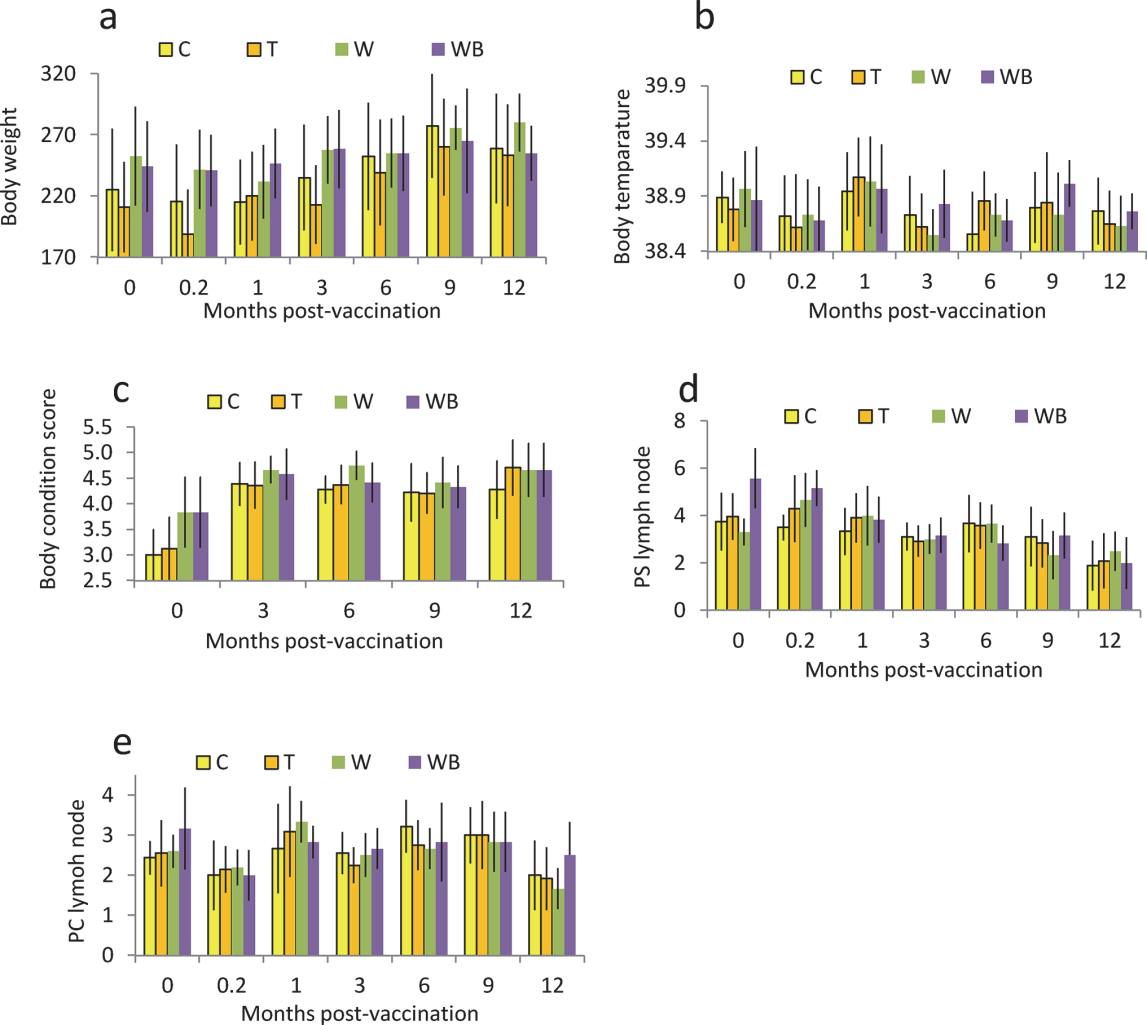
Mean (SD) body weight (a), body temperature (b), body condition score (c) and diameter of pre-scapular (d) and pre-crural (e) lymph nodes in Control (C), Treated (T), Webbed (W) and Webbed-boosted (WB) cattle. T, W and WB were treated with a single dose of the immunocontraceptive GonaCon, WB received a booster dose three months later. W and WB had their oviducts removed before the trial.

Body weight at vaccination day affected body weight during the rest of the trial, with animals heavier at the start remaining heavier throughout the trial (F_1,29 =_ 174.45, *P* < 0.001) (Fig 2A). Overall, body weight changed between vaccination day and six months post vaccination (F_3,87 =_ 24.28, *P* < 0.001) but varied differently between treatments (F_9,87 =_ 3.16, *P* < 0.004). However, there was no evidence of any overall treatment effect (F_3,29 =_ 1.03, *P* = 0.40).

Body temperature at vaccination day did not differ among groups (F_1,29 =_ 4.13, *P* = 0.051), although this effect was borderline (at the 5% significance level). Between vaccination day and six months post-vaccination, body temperature varied with time (F_3,87 =_ 8.38, *P* < 0.001) but there was no evidence that the trend differed between treatments (F_9,87 =_ 0.99, *P* = 0.45) (Fig 2B). Also, there was no evidence of any overall treatment effect (F_3,29 =_ 0.29, *P* = 0.83). Body condition score at vaccination day affected body condition score during the rest of the trial (F_1,28 =_ 6.74, *P* = 0.015), with animals with the highest scores on vaccination day maintaining high scores throughout the trial (Fig 2C). Between vaccination day and six months post-vaccination, body score did not vary with time (F_1,29 =_ 0.76, *P* = 0.39), and this was the case for all treatments (F_3,29 =_ 0.84, *P* = 0.48). There was no evidence of overall treatment effect (F_3,28 =_ 0.52, *P* = 0.68). The size of the PS lymph nodes on vaccination day affected the size of the PS lymph nodes during the rest of the trial (F_1,27 =_ 5.58, *P* = 0.026). The size of the PS lymph nodes did not differ among treatments overall (F_3,27 =_ 0.41, *P* = 0.75) but varied between vaccination day and six months post-vaccination, (F_3,73 =_ 6.96, *P* < 0.001). However, the trend of the size of the PS lymph node over time did not differ among treatments (F_9, 73 =_ 2.03, *P* = 0.060), although borderline (at the 5% significance level) (Fig 2D).

The size of the PC lymph nodes at vaccination day affected their size during the rest of the trial (F_1,25 =_ 9.69, *P* = 0.005). Between vaccination day and 6 months post vaccination the size of the PC lymph nodes varied with time (F_3,71 =_ 17.62, *P* < 0.001) but there was no evidence that this trend differed among treatments (F_9, 71 =_ 1.06, *P* = 0.40). Further, there was no evidence of any overall effect of the treatment (F_3,25 =_ 1.39, *P* = 0.27) (Fig 2E).

No differences in biochemistry and haematology were observed among groups at vaccination day (F_32,32 =_ 1.14, *P* = 0.35) and three months post-vaccination (F_32,30 =_ 1.46, *P* = 0.15) (Table 1).

**Table 1:** Mean (SD) hematological and biochemical parameters of cattle at vaccination and three months after vaccination.

	**Control**		**Treated**		**Webbed**	
Mean	Vaccination	3 month post-vaccination	Vaccination	3 month post-vaccination	Vaccination	3 month post-vaccination
Total Protein g/dL	7.96 (0.57)	8.57 (0.69)	8.12 (0.35)	8.48 (0.57)	7.96 (0.50)	8.20 (0.45)
Albumin g/dL	3.18 (0.29)	3.44 (0.45)	3.24 (0.38)	3.34 (0.31)	3.43 (0.42)	3.43 (0.26)
Globulin g/dL	4.80 (0.36)	5.12 (0.38)	4.88 (0.31)	5.12 (0.39)	4.54 (0.21)	4.77 (0.31)
SGOT/AST U/L	84.10 (10.59)	101.22 (18.40)	84.92 (20.74)	101.83 (23.90)	75.25 (7.33)	87.92 (24.33)
Gamma GT U/L	54.50 (32.96)	53.56 (27.40)	62.42 (65.85)	61.92 (95.09)	34.75 (11.26)	38.25 (20.52)
CPK U/L	307.30 (195.01)	243.56 (134.86)	258.25 (157.65)	283.25 (275.04)	272.67 (151.71)	227.67 (151.16)
Urea mg/dL	30.50 (5.06)	34.33 (4.72)	28.17 (7.00)	34.17 (3.66)	24.92 (2.91)	29.58 (4.34)
Sodium mmol/L	140.90 (3.60)	139.89 (2.42)	142.50 (2.78)	140.83 (1.64)	142.83 (2.66)	142.50 (1.09)
Potassium mmol/L	5.17 (0.43)	4.68 (0.42)	5.26 (0.45)	4.71 (0.40)	5.08 (0.50)	4.76 (0.41)
Cacium mg/dL	9.57 (0.52)	9.92 (0.43)	9.22 (0.55)	9.69 (0.43)	9.20 (0.42)	9.73 (0.27)
Phosforous mg/dL	7.33 (1.54)	7.77 (2.25)	7.54 (1.60)	8.64 (2.16)	6.35 (0.90)	8.10 (0.99)
Total WBC Count K/ul	9.73 (2.76)	11.50 (2.94)	9.91 (1.68)	10.58 (1.67)	8.58 (1.31)	9.75 (1.80)
Neutrophils K/ul	2.02 (0.89)	3.16 (1.58)	2.53 (0.78)	2.86 (1.12)	2.08 (0.85)	2.28 (0.86)
Lymphocites K/ul	6.23 (2.11)	6.53 (1.75)	5.77 (1.58)	6.03 (1.22)	5.32 (1.23)	5.75 (1.19)
Monocytes K/ul	0.39 (0.19)	0.50 (0.28)	0.41 (0.14)	0.51 (0.16)	0.26 (0.10)	0.39 (0.19)
Eosinophils K/ul	0.93 (0.34)	1.14 (0.29)	1.07 (0.50)	1.06 (0.40)	0.82 (0.39)	1.20 (0.68)
Neutrophils %	21.20 (8.35)	26.67 (8.15)	25.75 (6.99)	26.50 (7.99)	24.17 (8.45)	23.17 (7.09)
Lymphocites %	63.30 (9.43)	57.22 (8.79)	57.92 (8.96)	57.08 (8.47)	62.17 (9.69)	59.67 (10.90)
Monocytes %	4.00 (1.25)	4.44 (2.65)	4.08 (1.44)	5.00 (1.54)	2.92 (1.31)	4.00 (1.76)
Eosinophils %	10.00 (3.43)	10.33 (2.00)	11.17 (5.37)	10.25 (4.45)	9.58 (3.52)	11.92 (5.05)
RBC Count M/uL	8.07 (1.16)	8.43 (0.90)	8.31 (1.12)	7.88 (0.76)	8.45 (1.11)	8.05 (0.86)
Haemoglobin g/dL	13.01 (1.40)	13.91 (1.230	13.78 (1.69)	13.76 (1.21)	14.75 (1.74)	14.63 (1.48)
Haematocrit %	36.77 (3.95)	38.82 (3.35)	38.86 (4.77)	38.27 (3.30)	41.48 (5.240	40.44 (3.88)
MCV fL	46.09 (5.91)	46.26 (3.63)	47.06 (4.46)	48.88 (5.06)	49.18 (2.34)	50.34 (2.06)
MCH pg	16.30 (1.98)	16.56 (1.30)	16.70 (1.57)	17.58 (1.90)	17.47 (0.58)	18.18 (0.64)
MCHC g/dL	35.45 (1.38)	35.81 (1.07)	35.49 (0.72)	35.98 (0.87)	35.58 (1.140	36.15 (0.79)
RDW %	18.16 (1.07)	18.28 (1.18)	17.58 (1.57)	18.70 (1.73)	16.68 (1.130	17.78 (1.00)
Platelet count K/uL	220.00 (57.89)	205.78 (114.38)	242.58 (87.08)	255.92 (109.74)	193.83 (46.20)	193.67 (106.20)
MPV fL	9.99 (2.25)	10.49 (2.19)	10.72 (2.38)	9.16 (2.63)	10.60 91.090	10.88 (3.58

Controls were injected with a saline solution whilst Treated and Webbed (Groups T and W)cattle were injected with the immunocontraceptive GonaCon.

**Fig 3:**
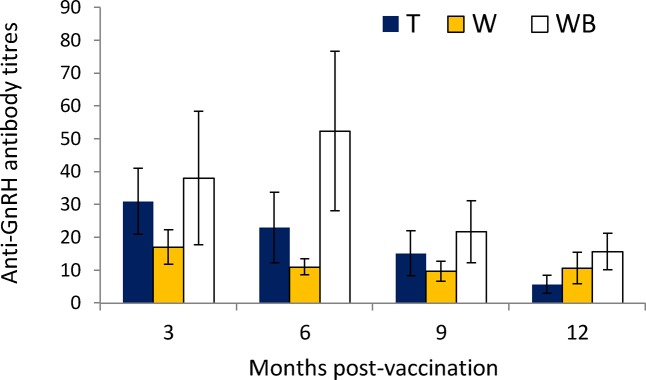
Mean (SD) anti-GnRH antibody titres for cattle treated with the immunocontraceptive vaccine GonaCon. All cattle received a single dose of GonaCon, WB received a booster dose three months later.

Out of 24 cattle treated with GonaCon, 23 had measurable anti-GnRH antibody titres; one animal in the T group had no detectable titres and was thus classified as a “non-responder” to the vaccine. The comparison in anti-GnRH antibody titres among Groups T, W, and WB showed that titres changed with time (F_3,63 =_ 20.44, *P* < 0.001) (Fig 3). The significant interaction between time and treatment (F_3,6 =_ 3.38, *P* < 0.019) indicated that the trend in titres over time differed among groups, as titres in animals that received a booster vaccination were shown to have declined less than those of cattle that received only a single dose; titres in the boosted cattle even increased at six months while none of the other treatments exhibited this trend. Despite infertile cattle having higher anti-GnRH titres at six months than cattle that became pregnant, the difference in anti-GnRH titres was not significant (Mann-Whitney U = 8.0, *P* = 0.19), possibly due to small sample size (8 not pregnant versus 4 pregnant animals).

Out of the 21 cattle that could become pregnant (n = 9 C, n = 12 T), the use of Kamar, carried out following the introduction of the bulls to the paddocks, suggested that 8 cattle in the C Group and 9 cattle in the T Group were cycling. The PAG test, carried out three months after the bulls had joined the females, indicated that all 9 Group C animals and 4 of the Group T females were pregnant. Pregnancy status was further confirmed by rectal palpation a month later. GonaCon significantly affected fertility in Group T cattle, rendering these animals significantly less likely to become pregnant than control animals (z = 3.113, *P* = 0.002).

Assuming that all the females that were found cycling through the use of the Kamar were also able to conceive, the Kamar confirmed cycling in all C animals except one that was found pregnant even though the Kamar did not indicate she had been mounted. In the T group, the Kamar suggested that nine cows were cycling: of these, only four became pregnant. The three animals in the T group that were not cycling according to the Kamar, did not become pregnant. The Kamar test correctly detected that 15 of 21 cows that would become pregnant were cycling while housed with the bulls. The results of the McNemar test indicated that the Kamar method may be a useful predictor of future pregnancy (χ² = 1.5, d.f. = 1, *P* = 0.22) in female cattle housed with intact bulls.

## Discussion

The results of this study showed that the immunocontraceptive GonaCon was safe when administered to cattle as a single dose or as prime and booster doses. No differences in any of the physiological and welfare indicators measured were found between treated and control animals, suggesting that GonaCon had no obvious negative side effects on this species. These findings are consistent with studies in other species such as wild boar that found no general side effect or specific injection site reaction in animals treated with GonaCon [23,24].

This study found that GonaCon was effective as a contraceptive, as 8 of the 12 treated animals (66.6%) became infertile, compared to all 9 control animals that became pregnant. Despite the relatively small sample size in the group treated with a booster dose, the results suggested that boosting is likely to increase the efficacy and duration of the contraceptive effect. The effectiveness of GonaCon to induce infertility in cows was comparable to that observed in other species where a single vaccination was successful in preventing pregnancy for multiple years. Comparisons between studies are hampered by the fact that the formulation of GonaCon differed in earlier studies as some of the constituents changed to improve the efficacy, decrease potential injection-site reaction, and reduce the cost of manufacturing the contraceptive [9, 17, 36].

Despite differences in formulation that might have affected some of the findings, GonaCon was found to reduce fertility in all ungulate species tested. A single 1800–2800 μg dose of GonaCon induced infertility in 93%, 64%, 53%, and 47% of captive mares in the four years following treatment respectively, compared to 0–25% of control animals that were infertile in the same period [25]. A field study showed that 1000 μg of GonaCon significantly reduced fertility in free-living horses for 3 years: 61%, 58%, and 69% of treated mares became infertile compared to 39%, 33%, and 24% of control mares [17]. A single 1000 μg dose of GonaCon rendered 91% of female wild boar infertile for at least 4–6 years, compared to 0% of the control sows [24]. Similarly, 88% and 47% of female white-tailed deer were infertile in the first two years after vaccination, respectively, compared with 15% and 0% of control animals [3].

Some studies have suggested that the dose of GonaCon might affect the anti-GnRH antibody titres and in turn the longevity of the contraceptive effect as well as the proportion of animals rendered infertile. However, other studies have found no evidence for dose-related efficacy. For instance, in elk anti-GnRH antibody titres were greater in females treated with a dose of 2000 μg than in females treated with 1000 μg of GonaCon. Interestingly, no difference in the rates of infertility between these groups was found in the three years following vaccination, with annual infertility of 90% -100% in treated animals of both groups compared to 0–27% in control females [37]. However, in feral pigs, vaccination with 2000 μg of GonaCon rendered 100% of the sows infertile whilst a 1000 μg dose caused infertility in 78% of the females [36]. The latter study and studies on wild boar [23,24], white-tailed deer [17], and feral horses [25] also suggested that higher anti-GnRH antibody titres were associated with greater rates of infertility.

The dose used in this study was consistent with that used previously in large animals, such as bison and horses. In female bison vaccinated with 1800 μg of GonaCon all 6 animals became infertile for at least one year whilst all 5 controls produced calves. Four of the 6 treated animals that were pregnant when injected with GonaCon, delivered healthy calves but did not reproduce the following season [38]. Similarly, all elk injected with 1500 μg of GonaCon when pregnant delivered healthy calves and then became infertile in the years following vaccination [27].

The only other trial with GonaCon in cattle was carried out on heifers in northern Australia: animals of considerably larger body mass (mean = 429 kg, SD = 28.3) than the cattle in this study were injected with a single dose of 3000 μg or with an initial dose of 2000 μg followed two months later by a 1000 μg booster [39]. The results showed that a single dose of GonaCon produced virtually no anti-GnRH response, whereas administration of a booster vaccination induced significant anti-GnRH antibody titres in 6 out of 9 heifers, and ovarian activity was suppressed for 330 days in 5 of 9 heifers [39]. One possible explanation for the lack of anti-GnRH antibody production following a single vaccine dose was that those cattle had not been previously exposed to *Mycobacterium avium*, one of the main components of the GonaCon adjuvant, which is otherwise thought to be ubiquitous in the rest of the world. The recommendations from the Australian study were that higher doses of GonaCon and administration of a booster should be tested in heifers to induce significant anti-GnRH antibody titres in these cattle. The results from the present study suggested that administration of a boost vaccination is likely to be more effective than treatment with a single dose and that the two doses employed as primer and booster were sufficient to induce anti-GnRH antibody titres that resulted in infertility in most animals.

Although the long-term infertility in cattle can only be assessed in a multi-year study, it is likely that some of the cattle that were rendered infertile with a single dose of GonaCon in this study will become fertile again due to the decrease in anti-GnRH antibodies. This is also confirmed by the finding that some animals appeared to be cycling, according to the Kamar, but did not become pregnant. These findings were consistent with the results of a study in white-tailed deer that found that the return of reproductive behaviour may occur years before the return of fertility [22]. This suggests that the follicular development and the estrogen concentration could be sufficient to support the expression of reproductive behaviour but insufficient to restore ovulation and fertility.

The decline in anti-GnRH antibody titres over time observed in this study is consistent with trends observed in other studies: in the years following vaccination with GonaCon, anti-GnRH antibody titres decrease and fertility is restored in some animals [3, 22, 36, 40]. Although the reversibility of the contraceptive effect can be a disadvantage when fertility control is used to manage overabundant populations of wildlife [9, 20], in other species, such as elephants and feral horses, temporary infertility might be desirable [18, 19]. The latter is likely to be the case for the Hong Kong cattle that many stakeholders would like keep in the area.

GonaCon is currently registered in the US as a contraceptive for white-tailed deer, feral horses and feral donkeys but it is also used as an experimental drug in other countries such as the UK [23, 24] and Australia [39, 41]. As the vaccine is broken down when ingested, GonaCon does not pose unacceptable risks to predators or human consumers of treated animals.

As pregnancy in cattle lasts approximately nine months, it is likely that a proportion of cattle will be pregnant at any time of the year. The fact that GonaCon does not appear to affect pregnancy is thus particularly important for field applications in cattle.

In conclusion, this study confirmed that GonaCon is a safe, effective option to induce infertility in cattle, thus warranting field applications for this species and consideration for other feral species in which population control becomes necessary, such as water buffaloes (*Bubalus bubalis*). Future field studies should evaluate effectiveness, feasibility and sustainability of immunocontraception at the population level to determine whether this method can be employed routinely to manage feral cattle in Hong Kong. If field studies confirm that cattle populations can be managed through immunocontraception, this method should also be explored for other contexts, such as in India, where religious beliefs prevent the use of lethal method, to mitigate the impact of cattle on human interests.

## References

[1] Rutberg AT, Naugle RE. Population effects of immunocontraception in white-tailed deer (*Odocoileus virginianus*). Wildl Res. 2008; 35: 494–501.

[2] Heydon M J. Wildlife conflict resolution: a review of problems, solutions and regulation in England. Wildl Res. 2010; 37: 731–748.

[3] Gionfriddo JP, Denicola AJ, Miller LA, Fagerstone KA. Efficacy of GnRH immunocontraception of wild white-tailed deer in New Jersey. Wildl Soc Bull. 2011; 35:142–148.

[4] Beringer J, Hansen LP, Demand JA, Sartwell J. Efficacy of translocation to control urban deer in Missouri: costs, efficiency, and outcome. Wildl Soc Bull. 2002; 30:767–774.

[5] Dickman AJ. Complexities of conflict: the importance of considering social factors for effectively resolving human-wildlife conflict. Anim Conserv. 2010; 13: 458–466.

[6] Sharp T, Saunders G. A model for assessing the relative humaneness of pest animal control methods Canberra: Australian Government Department of Agriculture, Fisheries and Forestry; 2008.

[7] McLeod SR, Saunders G. Fertility control is much less effective than lethal baiting for controlling foxes. Ecol Modell. 2014; 273:1–10.

[8] Duka T, Masters P. Confronting a tough issue: Fertility control and translocation for over-abundant Koalas on Kangaroo Island, South Australia. Ecol Manage Restor. 2005; 6:172–18.

[9] Fagerstone KA, Miller LA, Killian GJ, Yoder CA. Review of issues concerning the use of reproductive inhibitors, with particular emphasis on resolving human-wildlife conflicts in North America. Integr Zool. 2010; 1:15–30. 10.1111/j.1749-4877.2010.00185.x 21392318

[10] Delsink AK, Kirkpatrick J. Free-ranging African Elephant immunocontraception Cape Town: Trident Press; 2012.

[11] Fernando P, Leimgruber P, Prasad T, Pastorini J. Problem-elephant translocation: translocating the problem and the elephant? PloS ONE. 2012; 7(12), e50917 10.1371/journal.pone.0050917 23236404 PMC3517608

[12] Daszak P, Cunningham AA, Hyatt AD. Emerging infectious diseases of wildlife—Threats to biodiversity and human health. Science. 2000; 287: 443–449. 10642539 10.1126/science.287.5452.443

[13] Fernandez-de-Mera IG, Gortazar C, Vicente J, Hofle U, Fierro Y. Wild boar helminths: risks in animal translocations. Vet Parasitol. 2003; 115: 335–341. 12944047 10.1016/s0304-4017(03)00211-5

[14] Massei G, Quy RJ, Gurney J, Cowan DP. Can translocations be used to mitigate human-wildlife conflicts? Wildl Res. 2010; 37: 428–439.

[15] Fontúrbel FE, Simonetti JA. Translocations and human-carnivore conflicts: problem solving or problem creating?. Wildl Biol. 2011; 17: 217–224.

[16] McCall TC, Brown E. Movement and survival of translocated Rocky Mountain bighorn sheep in central Arizona. Desert Bighorn Council Transactions. 2011; 51: 11–16.

[17] Gray ME, Thain DS, Cameron EZ, Miller LA. Multi-year fertility reduction in free-roaming feral horses with single-injection immunocontraceptive formulations. Wildl Res. 2010; 37: 475–481.

[18] Kirkpatrick JF, Lyda RO, Frank KM. Contraceptive vaccines for wildlife: a review. Am J Reprod Immunol. 2011; 66: 40–50. 10.1111/j.1600-0897.2011.01003.x 21501279

[19] Druce HC, Mackey RL, Slowtow R. How immunocontraception can contribute to elephant management in small, enclosed reserves: Munyawana population. PLoS ONE. 2011; 6: 1–10. 10.1371/journal.pone.0027952 PMC3235100 22174758

[20] Massei G, Cowan P. Fertility control to mitigate human-wildlife conflicts: a review. Wildl Res. 2014; 41:1–21.

[21] Miller LA, Johns BE, Killian GJ. Immunocontraception of white-tailed deer with GnRH vaccine. Am J Reprod Immunol. 2000; 44: 266–274. 11125787 10.1111/j.8755-8920.2000.440503.x

[22] Killian G, Wagner D, Fagerstone K, Miller LA. Long-Term Efficacy and Reproductive Behavior Associated with GonaCon Use in White-Tailed Deer (*Odocoileus virginianus*). Proc 23rd Vert Pest Conf (Timm RM, Madon MB, Eds.) David: University of California; 2008. pp. 240–243.

[23] Massei G, Cowan DP, Coats J, Gladwell F, Lane JE, et al Effect of the GnRH vaccine GonaCon on the fertility, physiology and behaviour of wild boar. Wildl Res. 2008; 35: 1–8.

[24] Massei G, Cowan DP, Coats J, Bellamy F, Quy R, Brash M, et al Long-term effects of immunocontraception on wild boar fertility, physiology and behaviour. Wildl Res. 2012; 39: 378–385.

[25] Killian G, Thain D, Diehl NK, Rhyan J, Miller L. Four-year contraception rates of mares treated with single-injection porcine zona pellucida and GnRH vaccines and intrauterine devices. Wildl Res. 2008; 35: 531–539.

[26] Levy JK, Friary JA, Miller LA, Tucker SJ, Fagerstone KA. Long-term fertility control in female cats with GonaCon, a GnRH immunocontraceptive. Theriogenology. 2011; 76:1517–1525. 10.1016/j.theriogenology.2011.06.022 21835455

[27] Powers J, Baker DL, Davis TL, Conner MM, Lothridge AH, Nett TM. Effects of gonadotropin-releasing hormone immunization on reproductive function and behavior in captive female Rocky Mountain elk (*Cervus elaphus nelsoni*). Biol Reprod. 2011; 85: 1152–1160. 10.1095/biolreprod.110.088237 21753192

[28] Gionfriddo JP, Denicola AJ, Miller LA, Fagerstone KA. Health effects of GnRH immunocontraception of wild white-tailed deer in New Jersey. Wildl Soc Bull. 2011; 35:149–156.

[29] Yoder CA, Miller LA. Effect of GonaCon vaccine on black-tailed prairie dogs: Immune response and health effects. Vaccine. 2010; 29: 233–239. 10.1016/j.vaccine.2010.10.055 21055491

[30] Pei K J C, Lai Y C, Corlett RT, Suen KY. The larger mammal fauna of Hong Kong: species survival in a highly degraded landscape. Zool Stud. 2010; 49: 253–264.

[31] AFCD. Stray Cattle and Buffalo Management Plan; 2013. Available: http://www.afcd.gov.hk/english/quarantine/cattlebuffalo.html. **A**ccessed 2014 July 7.

[32] Ryan D. Cattle must have sound teeth NSW Agriculture AGfact AO.2.2, second edition Division of Agricultural Services; 1989 Available: ww.agric.nsw.gov.au. **A**ccessed 2014 July 7.

[33] Defra.Condition Scoring of dairy cows PB number 6492, Defra publications online; 2001. Available: https://www.gov.uk/government/uploads/system/uploads/attachment_data/file/69371/pb6492-cattle-scoring-diary020130.pdf. **A**ccessed 2014 July 7.

[34] Friedrich M, Holtz W. Establishment of an ELISA for measuring bovine pregnancy-associated glycoprotein in serum or milk and its application for early pregnancy detection. Reprod Domest Anim. 2010; 45: 142–146. 10.1111/j.1439-0531.2008.01287.x 19032429

[35] GenStat for Windows 16th Edition VSN International, Hemel Hempstead; 2013 Available: GenStat.co.uk. **A**ccessed 2014 Oct 7.

[36] Killian G, Miller L, Rhyan J, Doten H. Immunocontraception of Florida Feral Swine with a Single‐dose GnRH Vaccine. Am J Reprod Immunol. 2006; 55: 378–384. 16635212 10.1111/j.1600-0897.2006.00379.x

[37] Killian G, Kreeger TJ, Rhyan J, Fagerstone K, Miller LA. Observations on the Use of Gonacon in Captive Female elk (*Cervus elaphus*). J Wildl Dis. 2009; 45: 184–188. 19204347 10.7589/0090-3558-45.1.184

[38] Miller LA, Rhyan JC, Drew M. Contraception of bison by GnRH vaccine: a possible means of decreasing transmission of brucellosis in bison. J Wildl Dis. 2004; 40: 725–730. 15650090 10.7589/0090-3558-40.4.725

[39] D’Occhio MJ. GonaCon trial in heifers North Sydney: Meat & Livestock Australia Limited; 2013.

[40] Miller LA, Gionfriddo JP, Fagerstone KA, Rhyan JC, Killian GJ. The single-shot GnRH immunocontraceptive vaccine (GonaCon) in white-tailed deer: comparison of several GnRH preparations. Am J Reprod Immunol. 2008; 60: 214–223. 10.1111/j.1600-0897.2008.00616.x 18782282

[41] Snape MA, Hinds LA, Miller LA. Administration of the GnRH-targeted immunocontraceptive vaccine ‘GonaCon’ to the tammar wallaby, *Macropus eu*genii: side effects and welfare implications. In: Proc. 8th European Vertebrate Pest Management Conference, 114 Julius-Kühn-Archiv, 432, 2011. 10.5073/jka.2011.432.061.

